# Lysine β-hydroxybutyrylation as a drought-responsive epigenetic mark in rice

**DOI:** 10.1038/s41421-026-00868-7

**Published:** 2026-03-03

**Authors:** XueLu Wei, Zhengting Chen, Yuan Liu, Guiyu Xiao, Shengjun Guo, JiaDa Huang, Wen Ren, Xuan Ma, Xiaoyang Chen, Jisen Zhang, Qiutao Xu

**Affiliations:** 1 State Key Laboratory for Conservation and Utilization of Subtropical Agro-bioresources, College of Agriculture, Guangxi University, Nanning, Guangxi Zhuang Autonomous Region China; 2 State Key Laboratory of Rice Biology and Breeding, China National Rice Research Institute, Hangzhou, Zhejiang China; 3 Wuhan Bio-prolab Biotechnology Co. Ltd., Wuhan, Hubei China; 4 Beijing Key Laboratory of Maize DNA Fingerprinting and Molecular Breeding, Beijing Academy of Agriculture and Forestry Sciences, Beijing, China; 5 College of Agriculture, Ningxia University, Yinchuan, Ningxia Hui Autonomous Region China; 6 Anhui Province Key Laboratory of Crop Integrated Pest Management, Anhui Agricultural University, Hefei, Anhui China

**Keywords:** Plant molecular biology, Epigenetics

Dear Editor,

Protein post-translational modifications (PTMs), such as acetylation and methylation, are established as crucial epigenetic marks in eukaryotic cells. Recent advances in mass spectrometry (MS) have led to the discovery of novel PTMs, including crotonylation, β-hydroxybutyrylation, and lactylation^1^. Among them, lysine β-hydroxybutyrylation (Kbhb) has been well characterized in animal cells, particularly for its influence on energy metabolism. For instance, Kbhb inhibits *S*-adenosyl-l-homocysteine hydrolase (AHCY), a critical enzyme in the methionine cycle, associated with altered metabolite levels^2^. Notably, the detection of Kbhb in microbial species^3^ suggests that it may represent a conserved PTM across life forms. Recently, our study demonstrated that histone Kbhb regulates plant immunity by modulating the transcription of defense-related genes^4^. However, the presence and functional significance of Kbhb in plants, particularly under abiotic stress, remain largely unexplored. To address this gap, we conducted a comprehensive MS analysis of the β-hydroxybutyrylome in rice. Our results revealed that a substantial number of rice proteins were modified by Kbhb, further functional analysis demonstrated that Kbhb positively regulates drought stress responses in rice.

To identify specific proteins and modification sites of Kbhb in rice, Kbhb-modified peptides were enriched from rice seedlings and analyzed by MS (Fig. 1a). The identified peptides showed mass errors < 5 ppm and lengths of 7–20 amino acids, indicating high data quality (Supplementary Fig. S1). In total, 1222 Kbhb sites on 739 proteins were identified (Supplementary Dataset S1). Immunoblotting using a validated Kbhb-specific antibody^5^ confirmed the MS results and demonstrated the presence of Kbhb in other plant species (Fig. 1b). Approximately 34% of Kbhb-modified proteins contained multiple modification sites, including Rubisco large subunit and Heat shock protein 81 (Fig. 1c; Supplementary Fig. S2). These proteins were predominantly localized to the chloroplast (42.1%), cytoplasm (30.4%), and nucleus (11.6%) (Fig. 1d). Functional and pathway enrichment analyses revealed significant overrepresentation of metabolic processes, gene expression, translation, photosynthesis, and ATP metabolism, consistent with protein–protein interaction network analysis (Supplementary Figs. S3–S5). Motif analysis showed enrichment of leucine (L), phenylalanine (F), or tyrosine (Y) at the +1 position and glutamic acid (E) or glycine (G) at the −1 position flanking Kbhb sites, suggesting sequence preferences for Kbhb modification (Supplementary Fig. S6). Collectively, these data provide a comprehensive Kbhb proteome in rice, revealing the widespread occurrence of Kbhb modification on proteins and its potential regulatory roles in plant.

**Fig. 1:**
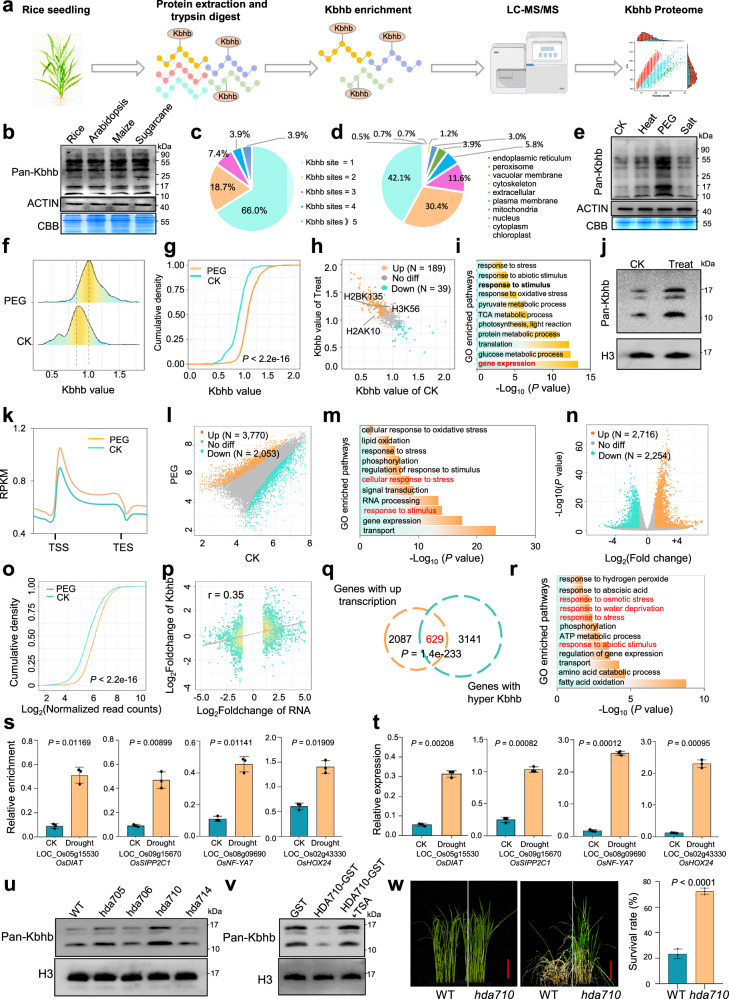
Identification and functional characterization of the Kbhb proteome in rice seedlings under drought stress. **a** Experimental workflow for identifying Kbhb-modified proteins. **b** Detection of Kbhb-modified proteins in various plant species using immunoblotting. Coomassie brilliant blue (CBB) staining and ACTIN were used as loading controls. **c** Distribution of the number of Kbhb sites per identified protein. **d** Pie chart of the subcellular localization of Kbhb-modified proteins. **e** Immunoblot detection of Kbhb levels in rice plants under untreated (CK), heat (42 °C), PEG (20%), and salt (150 mM) treatments. **f** Ridge plots of β-hydroxybutyrylation levels at individual lysine sites in PEG treated and CK rice plants. **g** Cumulative density plots of the β-hydroxybutyrylation levels at individual lysine sites in PEG treated and CK rice seedlings. **h** Scatter plot of proteins with significantly upregulated and downregulated β-hydroxybutyrylation levels at individual lysine sites in PEG-treated and CK rice seedlings. **i** GO pathway enrichment analysis of proteins (*n* = 189) with elevated Kbhb levels in PEG-treated plants. **j** Immunoblotting detection of histone Kbhb levels in CK and PEG-treated rice plants. **k** Metaplots showing the differential occupancy of histone Kbhb in rice plants subjected to PEG stress compared to the CK plants. **l** Scatter plot of ChIP-seq data showing the differential occupancy of histone Kbhb in rice plants treated with PEG compared to CK plants. **m** GO-enriched pathways of genes with significantly upregulated histone Kbhb levels. **n** Volcano plots of differential transcript levels in rice plants treated with PEG compared to CK plants. **o** Cumulative density plots of histone Kbhb levels in genes significantly upregulated in **n** in PEG-treated plants and CK plants. **p** Pearson correlation analysis between expression changes and histone Kbhb changes in PEG-treated plants relative to CK plants. **q** Overlap between genes with hyper-Kbhb levels and significantly increased expression under PEG stress. **r** GO-enriched pathway analysis of the genes (*n* = 629) in **q**. **s**, **t** ChIP–qPCR (**s**) and RT-qPCR (**t**) analyses of histone Kbhb modification and gene expression levels for four selected drought-responsive genes in PEG-treated and control (CK) plants. **u** Analysis of histone Kbhb levels in *hda705*, *hda706*, *hda710*, and *hda714* mutants compared to WT plants. **v** In vitro lysine de-β-hydroxybutyrylatase activity assay. **w** Phenotypes of *hda710* plants under PEG stress. Seedlings were subjected to PEG stress for 3 days, followed by a 7-day recovery period under normal growth conditions (25 °C). For **s**, **t**, **w** significant differences between the groups were analyzed using a Student’s *t*-test. ACTIN or histone H3 was used as the loading control in **b**, **e**, **j**, **u**, **v** as indicated. For **g**, **o** the *P* value was calculated using a two-sample Kolmogorov–Smirnov test. TSS, transcriptional start site; TES, transcriptional end site; TE, transposable elements.

Pathway analysis revealed significant enrichment of Kbhb-modified proteins in stress-response pathways (Supplementary Fig. S4), indicating a possible role of Kbhb in plant responses to abiotic stress. Consistently, immunoblotting showed a pronounced increase in global Kbhb levels in rice under polyethylene glycol (PEG)-induced drought stress (Fig. 1e), suggesting its potential role in drought stress regulation. Quantitative Kbhb proteomics, normalized to protein abundance, revealed an overall elevation of Kbhb following PEG treatment (Fig. 1f, g). Specifically, 189 Kbhb sites on 159 proteins were upregulated ( > 1.2-fold), whereas only 39 sites on 36 proteins were downregulated (Fig. 1h and Supplementary Dataset S2). Notably, three drought-induced sites were located on histones (H2AK10bhb, H2BK135bhb, and H3K56bhb), consistent with enrichment of gene expression-related pathways among proteins with increased Kbhb (Fig. 1i). Immunoblotting of histones and representative non-histone proteins further validated the dynamic changes of Kbhb proteome data under PEG treatment (Fig. 1j and Supplementary Figs. S7, S8).

Given increased histone Kbhb levels under drought stress and its reported role in transcription^5^, we next conducted chromatin immunoprecipitation followed by high-throughput sequencing (ChIP-seq) with a Kbhb-specific antibody to map its genomic distribution and assess its regulatory potential in rice. ChIP-seq analysis identified 27,682 Kbhb peaks corresponding to 18,351 genes in wild-type (WT) rice (Supplementary Dataset S3). ChIP–qPCR validation of regions with high and low Kbhb enrichment confirmed the reliability of the ChIP-seq data (Supplementary Fig. S9). Comparison with previously reported H3K9bhb profiles^4^ revealed a moderate correlation (*r* = 0.61), with substantial overlap between Kbhb- and H3K9bhb-marked genes (Supplementary Fig. S10). Genome-wide distribution analysis showed that Kbhb peaks were predominantly located within genic regions (Supplementary Fig. S11a). Among Kbhb-enriched genes, 91.5% were protein-coding (non-transposable element (TE)) genes, and 77% contained a single Kbhb peak (Supplementary Fig. S11b, c). Kbhb was preferentially enriched at transcription start sites (TSSs) of protein-coding genes rather than TE genes (Supplementary Fig. S11d). Correlation analyses with other histone modifications revealed that Kbhb was positively associated with active chromatin marks (H3K4ac, H3K9ac, H3K23ac, H4K5ac, and H3K4me3), but showed little or negative association with repressive marks (H3K9me2 and H3K9me3), suggesting a role in transcriptional activation (Supplementary Fig. S11e). Consistently, genes with higher expression levels exhibited stronger Kbhb enrichment (Supplementary Fig. S11f).

To identify genes regulated by histone Kbhb under drought stress, we compared histone Kbhb ChIP-seq profiles between PEG-treated and control (CK) rice plants. Metaplot analysis showed a global increase in histone Kbhb deposition under drought conditions (Fig. 1k). Differential analysis identified 3770 genes with increased Kbhb deposition and 2053 genes with decreased Kbhb deposition ( > 1.5-fold, *P* < 0.05; Fig. 1l and Supplementary Dataset S4). Gene Ontology (GO) analysis revealed that genes with elevated Kbhb levels were significantly enriched in stress-response pathways, supporting a regulatory role for histone Kbhb in drought adaptation (Fig. 1m). Consistent with β-hydroxybutyrate serving as the substrate for Kbhb, drought stress significantly increased cellular β-hydroxybutyrate levels (Supplementary Fig. S12). To assess transcriptional consequences, RNA-seq was performed on the same samples. Differential expression analysis identified 2716 upregulated and 2254 downregulated genes ( > 2-fold, *P* < 0.05) in PEG-treated plants relative to controls (Fig. 1n and Supplementary Dataset S5).

To characterize the relationship between histone Kbhb and gene transcription in rice, we first examined the correlation between changes in histone Kbhb levels and gene transcription in PEG-treated plants. Genes that were upregulated also exhibited increased deposition of Kbhb on histones (Fig. 1o). Furthermore, a moderate positive correlation (*r* = 0.35) was observed (Fig. 1p), further confirming the positive effect of Kbhb mediated regulation of gene transcription. To identify gene transcription directly regulated by histone Kbhb, an overlap between Kbhb-hyper and transcriptionally upregulated genes were performed. The analysis showed that a total of 629 genes with both increased Kbhb and changes in transcriptional regulation (Fig. 1q and Supplementary Dataset S6). GO pathway analysis of these genes revealed significant enrichment in biological processes related to stress responses, including water deprivation and osmotic stress pathways (Fig. 1r). Further examination of the genes that were both transcriptionally and Kbhb upregulated revealed 27 genes previously implicated in drought stress regulation (Supplementary Dataset S6). From this subset, we selected four genes, *SIPP2C1*, *OsHOX24*, *OsDIAT*, and *OsNF-YA7*^6,7^, to conduct ChIP–qPCR and RT-qPCR analyses (Fig. 1s, t and Supplementary Fig. S13), confirming the results obtained from ChIP-seq and RNA-seq datasets. Collectively, these results indicate that histone Kbhb is a histone mark strongly associated with active gene transcription and contributes to drought resilience in rice.

While histone Kbhb regulation by deacetylases is established in animals^8^, its regulation in plants remains poorly understood. To identify the specific regulator for Kbhb, we assayed the histone Kbhb levels in rice histone deacetylase mutant plants previously reported^4,9,10^. The results showed that mutations in *HDA710*, *SRT1*, *SRT2*, and *HDA705* led to a significant increase in histone Kbhb levels (Fig. 1u and Supplementary Fig. S14). To determine whether histone Kbhb regulated by these deacetylases contributes to the rice drought stress response, we analyzed histone Kbhb accumulation in the corresponding mutants under control and drought conditions. Notably, only the *hda710* mutant did not exhibit further increases in Kbhb levels under drought stress (Supplementary Fig. S14), suggesting that HDA710-regulated histone Kbhb plays an important role in drought resilience. To further validate the regulatory role of HDA710 on histone Kbhb, we expressed and purified recombinant HDA710 protein from *E. coli* and assessed its in vitro deacylation activity. The results showed that HDA710 efficiently removed both histone Kbhb and Kac modifications, but not Kcr or Kbu, and its activity was completely inhibited by Trichostatin A (TSA), a well-known histone deacetylase inhibitor (Fig. 1v and Supplementary Fig. S15). Since HDA710 also possesses histone deacetylase activity, we detected Kac levels (with Ksu and Kcr as controls) under control and drought conditions. Kac levels were further increased in the *hda710* mutant under drought stress (Supplementary Fig. S16), indicating that HDA710-regulated Kac is unlikely to be a major contributor to drought adaptation. To further validate the role of histone Kbhb in drought response, we examined the *hda710* mutant phenotype. Mutation of *HDA710* significantly enhanced the survival rates of rice plants under drought stress (Fig. 1w and Supplementary Fig S17). To investigate the role of HDA710 in regulating histone Kbhb during drought stress, we analyzed four known drought-responsive genes in both *hda710* and WT plants. Our analysis revealed that these genes exhibited significantly elevated levels of both Kbhb enrichment and transcription in the *hda710* mutant compared to WT (Supplementary Fig. S18). Thus, HDA710 is a key regulator of histone Kbhb in rice, and likely controls drought-responsive gene transcription, though effects from Kac remain possible.

In this study, a total of 1222 Kbhb sites on 739 proteins were identified, revealing widespread Kbhb modification in rice. Unlike animals, where Kbhb is predominantly nuclear, rice Kbhb-modified proteins were enriched in cytoplasmic metabolic pathways, indicating evolutionary diversification. Importantly, Kbhb-modified proteins were significantly enriched in stress-response pathways, suggesting a role in environmental adaptation. Genome-wide profiling demonstrated that histone Kbhb acts as an active chromatin mark in plants, with drought stress inducing its deposition at drought-responsive genes to promote transcription. We further identified HDA710 as a histone Kbhb deacylase that regulates drought tolerance, likely by modulating histone Kbhb levels and reprogramming stress-responsive gene expression. HDA710 is functionally distinct from previously characterized Kbhb erasers (SRT1, SRT2, and HDA705)^4^, which target metabolic or defense-related pathways, highlighting specialization among Kbhb erasers in rice. Although HDA710-mediated de-Kbhb plays a key role in drought adaptation, contributions from its deacetylase activity cannot be excluded. Moreover, the identity of the Kbhb writer under drought stress remains unknown. In animals, p300 has been established as a Kbhb writer^8^. Based on sequence homology, rice p300 homologs, particularly HAC703, are promising candidates, as HAC703 is markedly upregulated under drought conditions^11^. It is also critical to acknowledge that the current evidence remains largely correlative; future studies should use direct approaches, such as dCas9-based epigenetic editing, to test the causal roles of Kbhb at specific loci. Collectively, our study expands the functional landscape of Kbhb in plants, provides a mechanistic framework for understanding its role in stress adaptation, and opens new avenues for leveraging this epigenetic pathway in crop improvement.

## Supplementary information

Supplementary Information

Supplementary Dataset S1 Kbhb proteins identified in rice seedlings

Supplementary Dataset S2 Proteins with significantly changed Kbhb levels in PEG treated plants in comparison to CK

Supplementary Dataset S3 Kbhb enriched regions indentified in wild type rice plants

Supplementary Dataset S4 Genes with significantly changed Kbhb deposition in PEG treated plants in comparison to CK

Supplementary Dataset S5 Genes with significantly up- and down-regulation in PEG treated plants in comparison to CK

Supplementary Dataset S6 Genes with significantly upregulated Kbhb levels and transcription

## Data Availability

Data have been deposited as follows: MS data in PRIDE (PXD059711, PXD059709); RNA-seq and ChIP-seq data in NCBI SRA (PRJNA1282469).
