# Supplementary material for: Lysine β-hydroxybutyrylation as a drought-responsive epigenetic mark in rice
Source: Cell Discov. 2026 Mar 3;12:14. doi: 10.1038/s41421-026-00868-7 (PMC12953871; doi:10.1038/s41421-026-00868-7)
Supplement: Supplementary file 1 — Supplementary Information [file 41421_2026_868_MOESM1_ESM.pdf]

Supplementary information for

Lysine β-Hydroxybutyrylation as a Drought-Responsive Epigenetic Mark in Rice

XueLu Wei[#1], Zhengting Chen[#2], Yuan Liu[#3], Guiyu Xiao[1], Shengjun Guo[1], JiaDa Huang[1], Wen Ren[4], Xuan Ma[5], Xiaoyang Chen[6], Jisen Zhang[1]*, Qiutao Xu[1]*

Affiliations:

[1]State Key Laboratory for Conservation and Utilization of Subtropical Agro-bioresources, College of Agriculture, Guangxi University, Nanning 530004, China

[2]State Key Laboratory of Rice Biology and Breeding, China National Rice Research Institute, Hangzhou 311400, China

[3]Wuhan Bio-prolab Biotechnology Co., LTD， Wuhan, 430070, China

[4]Beijing Key Laboratory of Maize DNA Fingerprinting and Molecular Breeding, Beijing Academy of Agriculture and Forestry Sciences, Beijing 100097, China

[5]College of Agriculture, Ningxia University, Yinchuan,750021, China

[6]Anhui Province Key Laboratory of Crop Integrated Pest Management, Anhui Agricultural University, Hefei 230036, China

[#]These authors contribute equally.

*Corresponding authors: Jisen Zhang and Qiutao Xu

Email: zjisen@126.com (J.Z.); qiutaoxu@gxu.edu.cn (Q.X.)

This PDF file includes:

Materials and Methods

References

Supplementary figures

Materials and Methods

Plant materials and treatment

In this study, we utilized CRISPR/Cas9-generated histone deacetylase mutants, as previously reported [1-3]. For phenotypic analysis, the plants were grown hydroponically in liquid Yoshida's solution, and stress treatment was applied to seedlings at the early stages of development. Osmotic stress was induced by adding 20% PEG6000 to the nutrient solution for 3 days. Control seedlings were maintained under standard conditions of 25 °C with a 14-hour light period and 25 °C with a 10-hour dark period. Following stress treatments, the seedlings were returned to normal conditions for 7 days to assess survival. Survival rates were determined by calculating the percentage of viable seedlings (live plants/total plants × 100%) after recovery, with three biological replicates (n = 40 per replicate).

Histone extraction

Histone extraction was carried out using the Histone Extraction Kit (Epigentek USA, OP-0006-100). Briefly, rice samples were ground in liquid nitrogen and suspended in 1X Pre-Lysis Buffer. The samples were centrifuged at 15,000 g for 5 minutes at 4 °C, and the pellets were collected. The pellets were then lysed in Lysis Buffer for 30 minutes at 4 °C, followed by centrifugation at 12,000 rpm for 5 minutes at 4 °C. The supernatant was transferred to a new tube, and Balance Buffer was added. The final enriched histones were analyzed with the following primary antibodies: anti-β-hydroxybutyryllysine (1:1000, PTM-1201, PTM Biolabs), anti-acetyllysine (1:1000, PTM-102, PTM Biolabs), anti-anti-crotonyllysine (1:1000, PTM-501, PTM Biolabs), anti-butyryllysine (1:1000, PTM-301RM, PTM Biolabs).

Protein Kbhb level assay

For the Kbhb level assay on none-histone proteins, total proteins were extracted using a buffer containing 50 mM HEPES-KOH (pH 7.5), 150 mM NaCl, 1 mM EDTA, 1% Triton X-100, 0.1% sodium deoxycholate, and 0.1% SDS. The extracted proteins were

then incubated with protein A magnetic beads (Thermo Fisher Scientific, 10001D) pre-coated with the corresponding target protein antibody for 5 h at 4 °C. After four washes with PBST buffer, the immunoprecipitated proteins were collected and boiled at 95 °C. For the Kbhb level assay on histones, histones were first extracted using a Histone Extraction Kit (for detailed steps, see Histone extraction) and then boiled at 95 °C. The resulting proteins were separated on 12–15% SDS–PAGE and transferred onto a PVDF membrane. The membrane was probed with the following primary antibodies: anti-β-hydroxybutyrylated lysine (1:1000, PTM-1201, PTM Biolabs), anti-RPS3 (1:1000, Abmart, PS18836), anti-GAPDH (1:1000, Abcam, ab9485), and anti-Rubisco L (1:1000, Abmart, MA9210S).

Measurement of β-hydroxybutyrate Content

The β-hydroxybutyrate content was determined using a commercial β-hydroxybutyrate Content Assay Kit (AKCO027M, Beijing Boxbio Science & Technology Co.,Ltd.) following the manufacturer's instructions. Briefly, 0.1 g of rice leaf tissue was ground into fine powder in liquid nitrogen and extracted with prechilled phosphate-buffered saline (PBS, pH 7.4). The homogenate was centrifuged at 12,000 × g for 10 min at 4 °C, and the supernatant was collected for analysis. The working solution was freshly prepared by mixing Reagents I, II, and III at a volume ratio of 85:4:1, and preincubated at 37 °C for 15 min before use. The extracted sample was mixed with the working solution and incubated at 37 °C for 10 min, followed by addition of Reagent VI and further incubation at 37 °C for 20 min. Absorbance was measured at 450 nm using a microplate reader. The β-hydroxybutyrate concentration was calculated based on the standard curve.

***In vitro* de-Kbhb assay**

First, histones were extracted following a modified acid extraction protocol reported in a previous study [4]. Approximately 2 g of frozen plant tissue was ground in liquid nitrogen and homogenized in 20 mL of pre-chilled extraction buffer (10 mM Tris-HCl, pH 7.5, 2 mM EDTA, 0.25 M HCl, 5 mM DTT, and protease inhibitor cocktail

[Roche]). The homogenate was filtered through a single layer of Miracloth and centrifuged at 12,000 × g for 10 min at 4 °C. The supernatant (16 mL) was mixed with 4 mL of 100% trichloroacetic acid (TCA) and centrifuged at 17,000 × g for 30 min at 4 °C. The resulting pellet was washed three times with pre-chilled (–20 °C) acetone, air-dried until powder-like, and stored at –80 °C for *in vitro* de-Kbhb assay. Next, *in vitro* de-β-hydroxybutyrylation assays were conducted using GST-tagged HDA710 proteins, which were expressed and purified from *E. coli*. The reactions were performed at 37 °C for 6 hours in a buffer composed of 2.5 μg histone proteins, 50 mM Tris-HCl (pH 8.5), 137 mM NaCl, 2.7 mM KCl, 1 mM $MgCl_2$, and 1 mM dithiothreitol (DTT). Following the reaction, the products were analyzed by western blotting using an anti-Kbhb antibody (1:1000, PTM-1201, PTM Biolabs).

qPCR and ChIP-qPCR

Total RNA was extracted from rice leaves using TRIzol reagent (Invitrogen). First-strand cDNA was synthesized with the HiScript II Q RT SuperMix for qPCR kit (Vazyme Biotech, R223-01). Real-time PCR was performed using the 2 × SYBR Mix (Vazyme Biotech, Q712-02) on a real-time PCR system (Applied Biosystems). ACTIN1 was used as the internal control gene. Gene expression levels were calculated based on three independent biological replicates.

ChIP assays were performed on rice samples using a pan anti-Kbhb antibody (PTM-1201; PTM Biolabs). Briefly, one gram of rice flowers was cross-linked with 1% formaldehyde for chromatin extraction. After sonication, the chromatin fragments were incubated overnight at 4 °C with antibody-coated beads. Following extensive washing, the cross-linking was reversed, and the immunoprecipitated chromatin was recovered for qPCR analysis. Three independent biological replicates were performed.

RNA-seq and data analysis

RNA was isolated using TRIzol reagent (Invitrogen) as previously described[5]. RNA-seq libraries were prepared using the Illumina TruSeq RNA Sample Preparation Kit and sequenced on the Illumina HiSeq 2000 platform using the paired-end 150 bp

(PE150) method.

Raw RNA-seq data were filtered using FastP (v0.232) to remove contaminants and low-quality reads. Clean reads were mapped to the rice genome (MSU 7.0) using Subjunc (v2.0.14), and read counts were calculated with FeatureCounts (v2.0.3). Genes with a *p-value* < 0.05 and a fold change > 2 in PEG-treated samples were considered differentially expressed.

ChIP-Seq and data analysis

Rice samples were cross-linked with 1% formaldehyde (F8775, Sigma-Aldrich) for 30 minutes, then ground into a powder using liquid nitrogen. The powdered samples were extracted using lysis buffer (10 mM Tris-HCl, pH 8.0, 0.4 M sucrose, 0.1 mM PMSF, 10 mM MgCl2, 5 mM β-mercaptoethanol, and a protease inhibitor cocktail). The resulting mixture was passed through a double layer of Miracloth (475855, Millipore) and centrifuged at 4,000 g for 20 minutes at 4 °C. The chromatin pellet was then washed five times with washing buffer (0.25 M sucrose, 1% Triton X-100, 10 mM Tris, pH 8.0, 10 mM $MgCl_2$, 0.1 mM PMSF, 5 mM β-mercaptoethanol, and a protease inhibitor cocktail) and resuspended in 300 μL of nucleus lysis buffer (50 mM Tris-HCl, pH 8.0, 10 mM EDTA, 1% [w/v] SDS). The chromatin was sonicated to fragments of 200–500 bp, with 20 μL set aside as input, and then immunoprecipitated using an anti-Kbhb antibody (PTM-1201, PTM Biolabs). The immunoprecipitated chromatin was subjected to sequential washes and eluted in elution buffer (10 mM EDTA, 50 mM Tris-HCl, pH 7.5, 1% SDS) at 65 °C for 15 minutes with shaking at 800 rpm. The eluted chromatin was reverse-crosslinked overnight at 65 °C in 5 M NaCl, followed by purification with chloroform and alcohol precipitation.

Purified DNA fragments were used to construct libraries according to the protocol provided in the Illumina TruSeqChIP Sample Prep Set A kit and were sequenced using paired-end 150 bp reads (PE150) on the Illumina HiSeq 2000 platform. For data analysis, raw sequencing reads were first cleaned using FastP (v0.232) to remove low-quality reads and adapter sequences. The cleaned reads were then aligned to the

rice genome using Bowtie2 (v2.3.5.1) with default parameters. Duplicate reads were filtered out using Samtools (v1.9), and histone modification peaks were called with MACS (v2.2.7.1) using the default settings (-f BAMPE -B -q 0.05). Wiggle files (BPM normalization) generated by deepTools (v2.5.3) were visualized in the Integrative Genomics Viewer (IGV, v2.3.88). Differential peaks for histone modifications were identified using DiffBind (v3.5) with default parameters, and peak annotation was performed with Homer's annotatePeaks.pl script (v4.11). Gene ontology (GO) enrichment analysis was carried out using the clusterProfiler package (v4.10.0). Ridge plots, scatter plots, and heatmaps were created using Tbtools (v0.6) and R (v3.5), respectively.

Protein Extraction

Samples were ground in liquid nitrogen into a fine powder. Four times the volume of phenol extraction buffer (containing 10 mM DTT, 1% protease inhibitors, 50 μM PR-619, 3 μM TSA, 50 mM NAM) was added to samples, followed by sonication for lysis. An equal volume of Tris-buffered phenol was added, and the mixture was centrifuged at 5,500 g for 10 min at 4 °C. The supernatant was collected, and five times the volume of 0.1 M ammonium acetate/methanol was added for precipitation overnight. The precipitate was washed with methanol and acetone, and finally, the pellet was kept for further analysis.

LC-MS/MS

Protein pellets were dissolved in 200 mM TEAB, and dispersed by sonication. Then trypsin was added at a ratio 1:50 trypsin-to-protein mass ratio and digested overnight. Dithiothreitol (DTT) was added to a final concentration of 5 mM, and the sample was reduced at 56 °C for 30 min. Iodoacetamide (IAA) was then added to a final concentration of 11 mM, and the sample was incubated in the dark at room temperature for 15 min. Finally, the peptides were desalted by Strata X SPE column. The peptides were then dissolved in solvent A and separated using the NanoElute UHPLC system. The mobile phase consisted of solvent A (0.1% formic acid, 2%

acetonitrile/in water) and solvent B (0.1% formic acid in acetonitrile). The gradient was set as follows: 0-70 min, 6%-24% B; 70-82 min, 24%-35% B; 82-86 min, 35%-80% B; 86-90 min, 80% B. The flow rate was maintained at 450 nL/min. After separation, the peptides were ionized in the capillary ion source and analyzed using the timsTOF Pro mass spectrometer. The ion source voltage was set to 1.7 kV, and both precursor and fragment ions were detected with high-resolution TOF. The MS/MS scan range was set to 100-1700 m/z. Data were acquired in parallel accumulation-serial fragmentation (PASEF) mode, with one MS scan followed by ten PASEF mode acquisitions of MS/MS spectra for precursor ions with charges of 0-5. The dynamic exclusion time for repeated scans of precursor ions was set to 30 s to avoid duplicate scans. Proteins that exhibited less than 1.2-fold change in Kbhb levels in both replicates were annotated as decreased.

References

1 Ullah F, Xu Q, Zhao Y, Zhou DX. Histone deacetylase HDA710 controls salt tolerance by regulating ABA signaling in rice. *J Integr Plant Biol* 2020.

2 Chen X, Xu Q, Duan Y *et al.* Ustilaginoidea virens modulates lysine 2-hydroxyisobutyrylation in rice flowers during infection. *J Integr Plant Biol* 2021; **63**:1801-1814.

3 Xu Q, Liu Q, Chen Z *et al.* Histone deacetylases control lysine acetylation of ribosomal proteins in rice. *Nucleic Acids Res* 2021; **49**:4613-4628.

4 Xu Q, Yue Y, Liu B *et al.* ACL and HAT1 form a nuclear module to acetylate histone H4K5 and promote cell proliferation. *Nat Commun* 2023; **14**:3265.

5 Zhang J, Qi Y, Hua X *et al.* The highly allo-autopolyploid modern sugarcane genome and very recent allopolyploidization in Saccharum. *Nat Genet* 2025; **57**:242-253.

Supplementary Figures

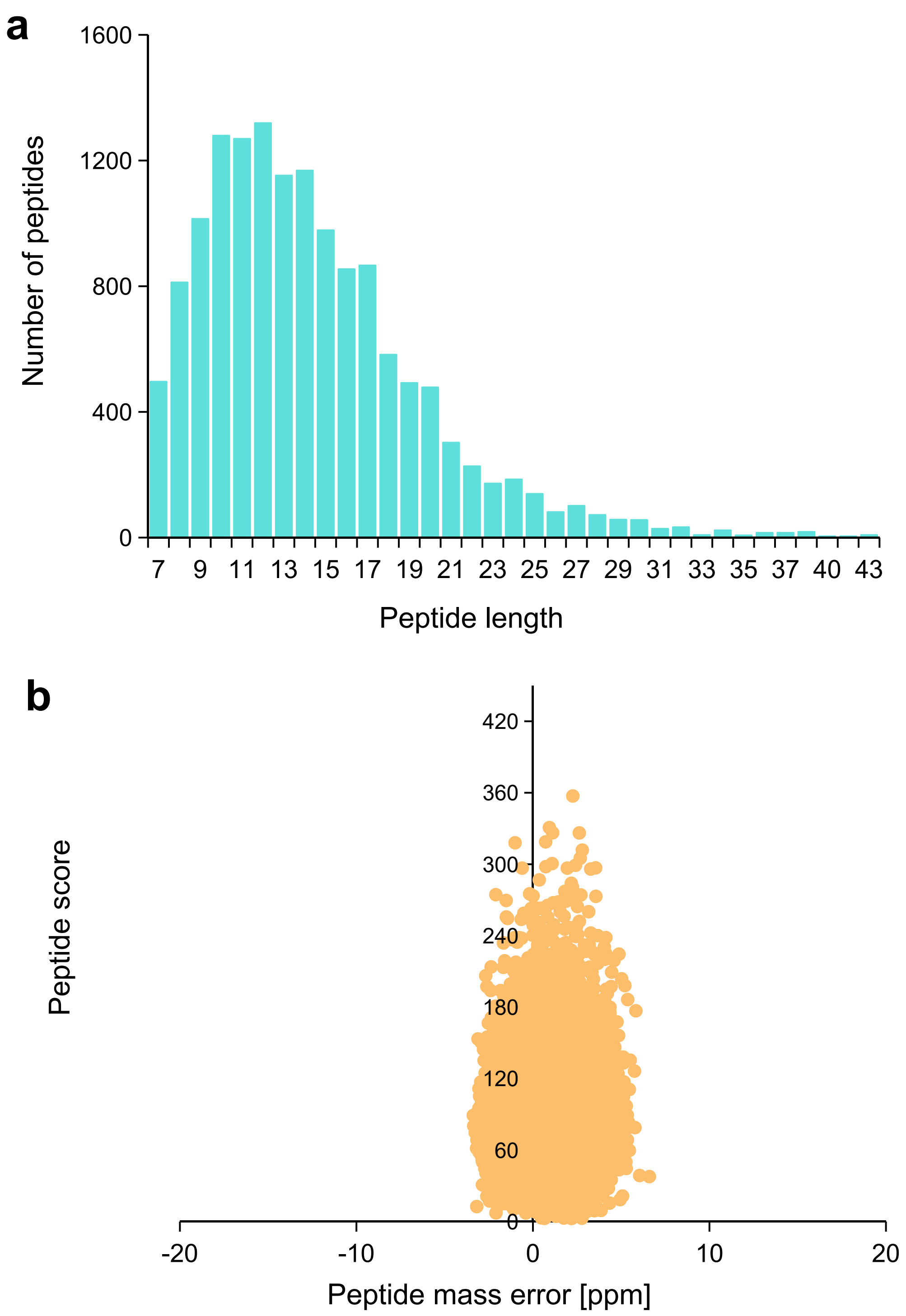

Supplementary Fig. S1. Quality control of mass spectrometry (MS) data. a Peptide length distribution of Kbhb site-containing peptides detected by mass spectrometry. **b** Mass error distribution of the identified Kbhb peptides.

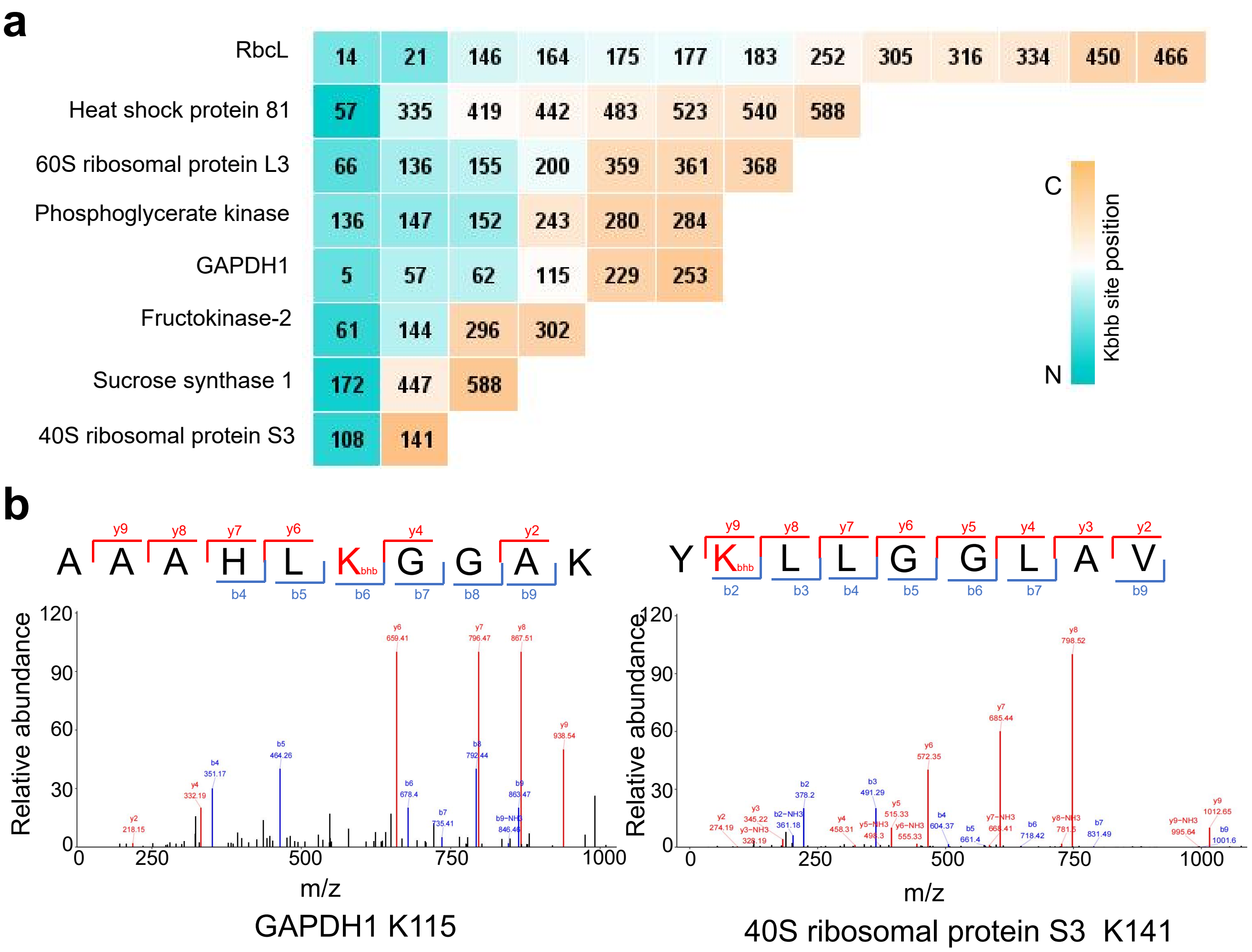

Supplementary Fig. S2. Kbhb sites analysis. a Representative Kbhb proteins detected in rice seedlings. Numbers within each square indicate the lysine residue modified by Kbhb. Kbhb positions near the N- and C-termini are highlighted in cyan and yellow, respectively. **b** Left: MS/MS spectra of the tryptic peptide AAAHLK(bhb) GGAK from GAPDH1 Kbhb peptide. Right: MS/MS spectra of the tryptic peptide YK(bhb) LLGGLAV from the 40S ribosomal protein S3 Kbhb peptide. RbcL, ribulose-1,5-bisphosphate carboxylase/oxygenase large subunit; GAPDH, glyceraldehyde 3-phosphate dehydrogenase.

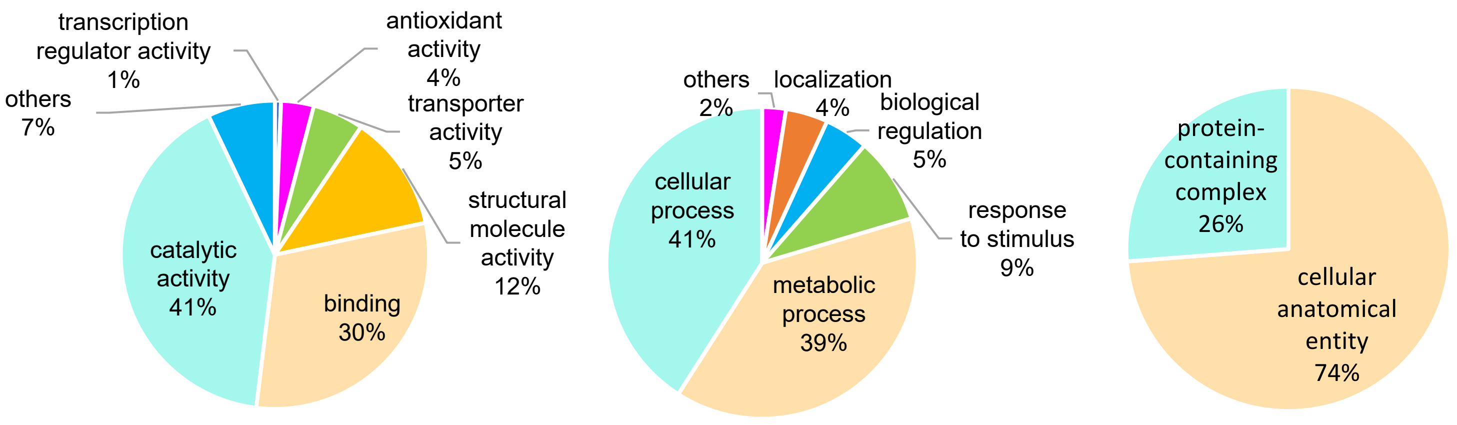

Supplementary Fig. S3. Functional classification of Kbhb-modified proteins, categorized by biological processes, molecular functions, and cellular components.

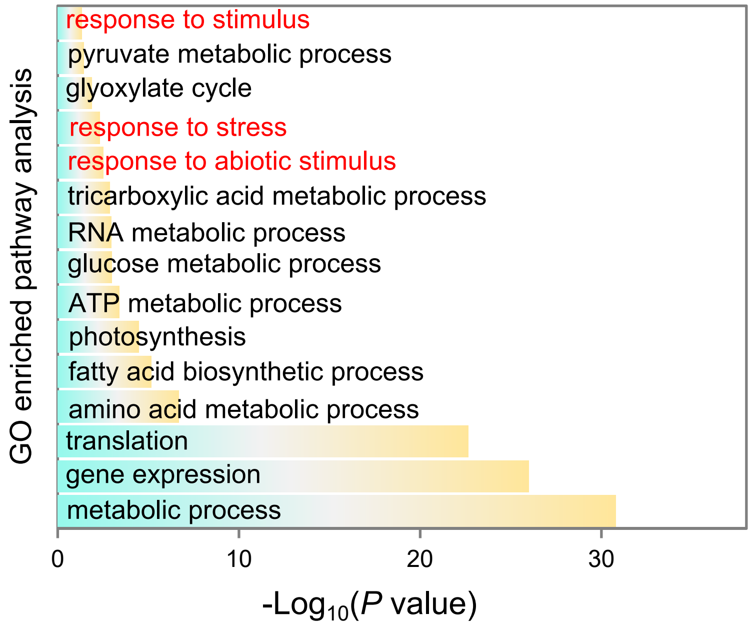

Supplementary Fig. S4. GO biological pathway enrichment analysis of Kbhb-modified proteins.

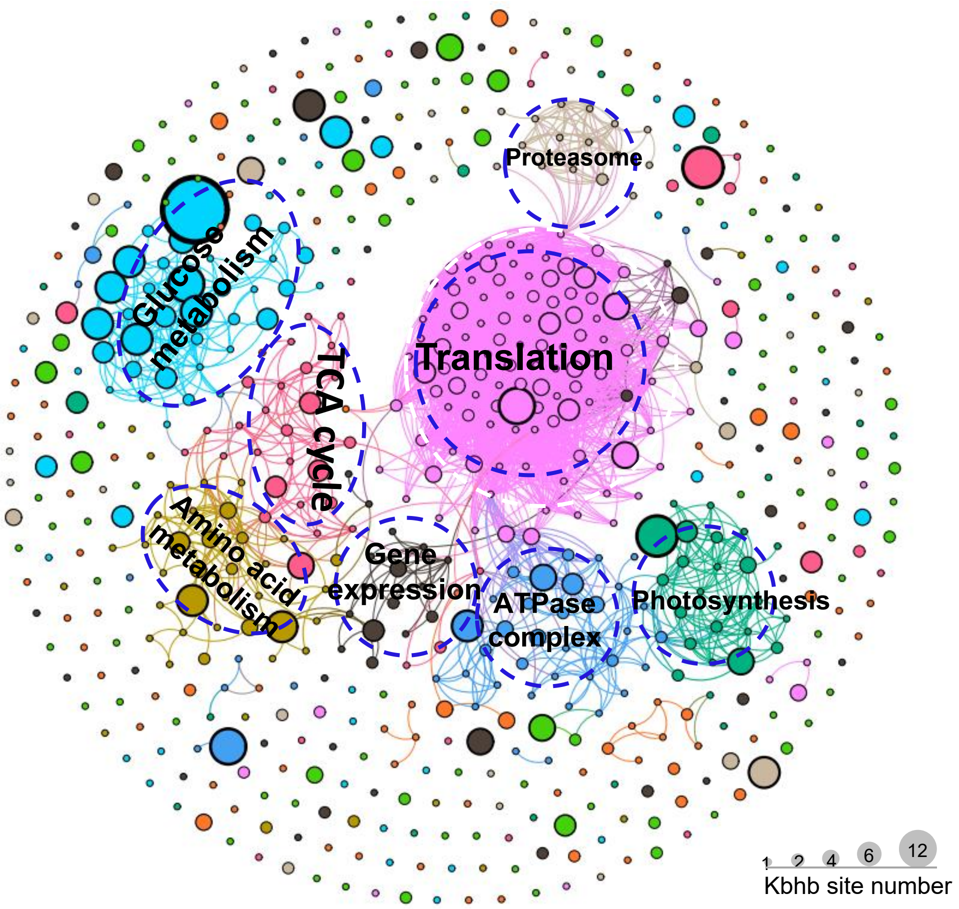

Supplementary Fig. S5. Protein–protein interaction (PPI) map of Kbhb-modified proteins identified in rice seedlings. Different colors represent proteins belonging to distinct clusters.

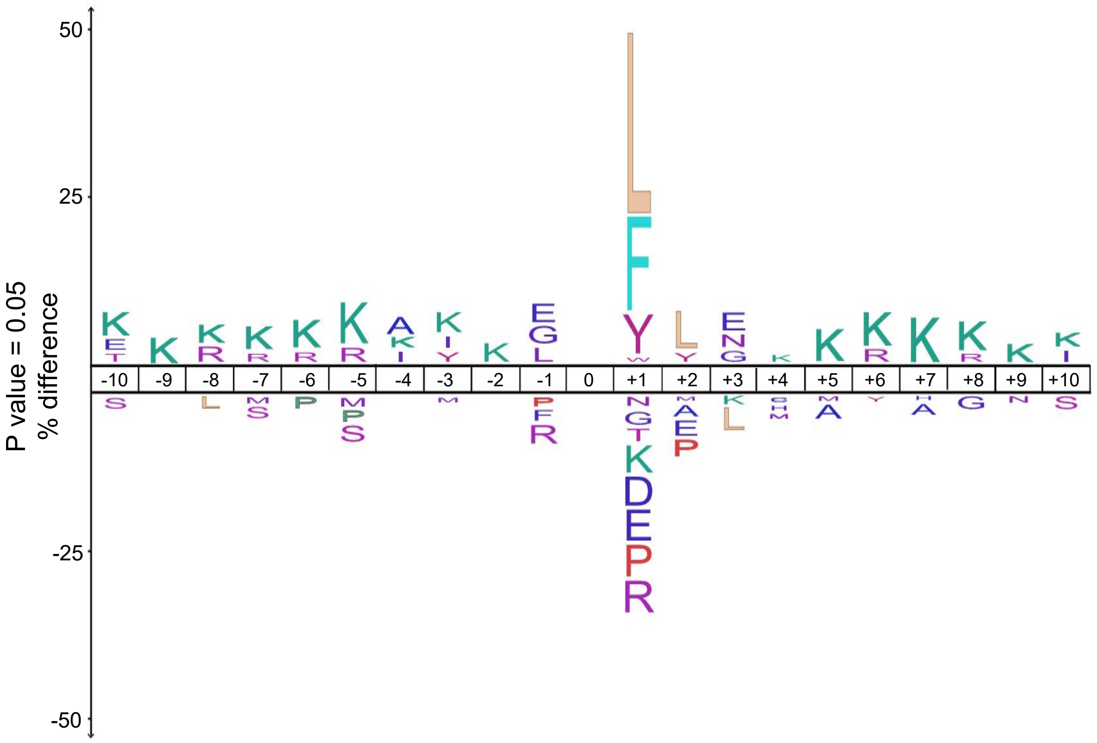

Supplementary Fig. S6. Motif analysis of Kbhb-modified proteins. The size of each amino acid indicates the variation between its observed frequency in the experiment and its expected frequency in the reference set. The *P-value* for each amino acid at every position was determined by comparing its experimental frequency with that in the reference set using Fisher’s exact test.

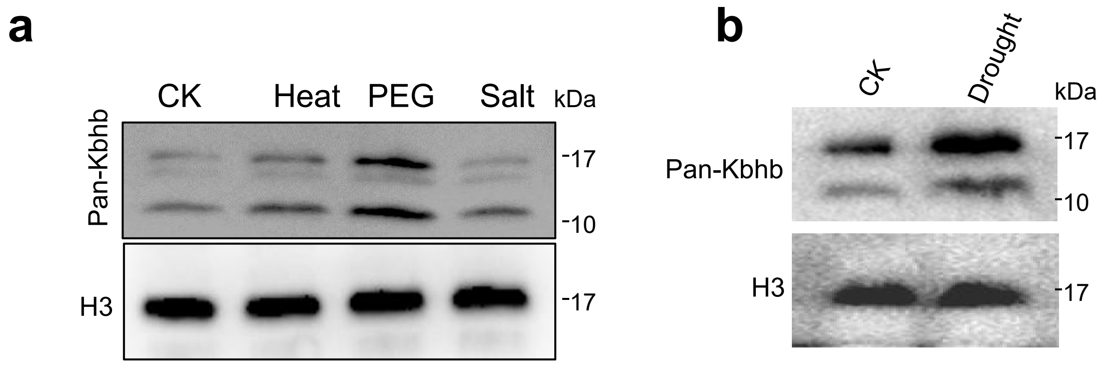

Supplementary Fig. S7. Assay of Histone Kbhb levels in rice plants under various stresses. a Histone Kbhb levels in rice under heat, PEG, salt, and control (CK) conditions . **b** Histone Kbhb levels in rice under soil drought and CK conditions. Histone H3 was used as the loading control.

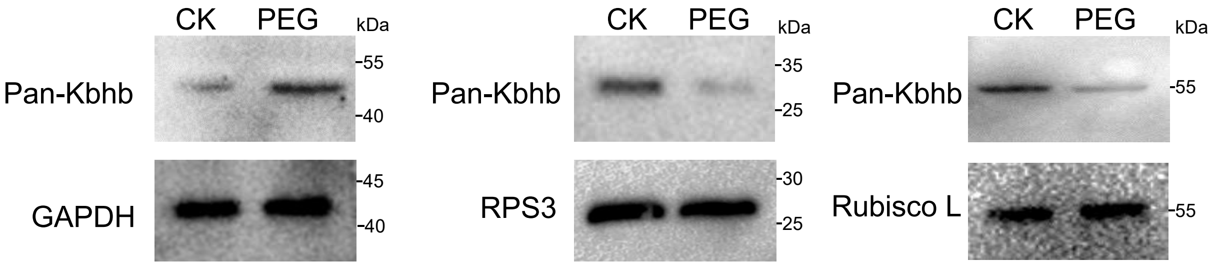

Supplementary Fig. S8. Dynamic changes in Kbhb levels of glyceraldehyde-3-phosphate dehydrogenase (GAPDH)，40S ribosomal protein S3 (RPS3)，and ribulose bisphosphate carboxylase large chain (Rubisco L) in untreated (CK) and PEG-treated rice plants. GAPDH, RPS3, and Rubisco L were used as the loading controls.

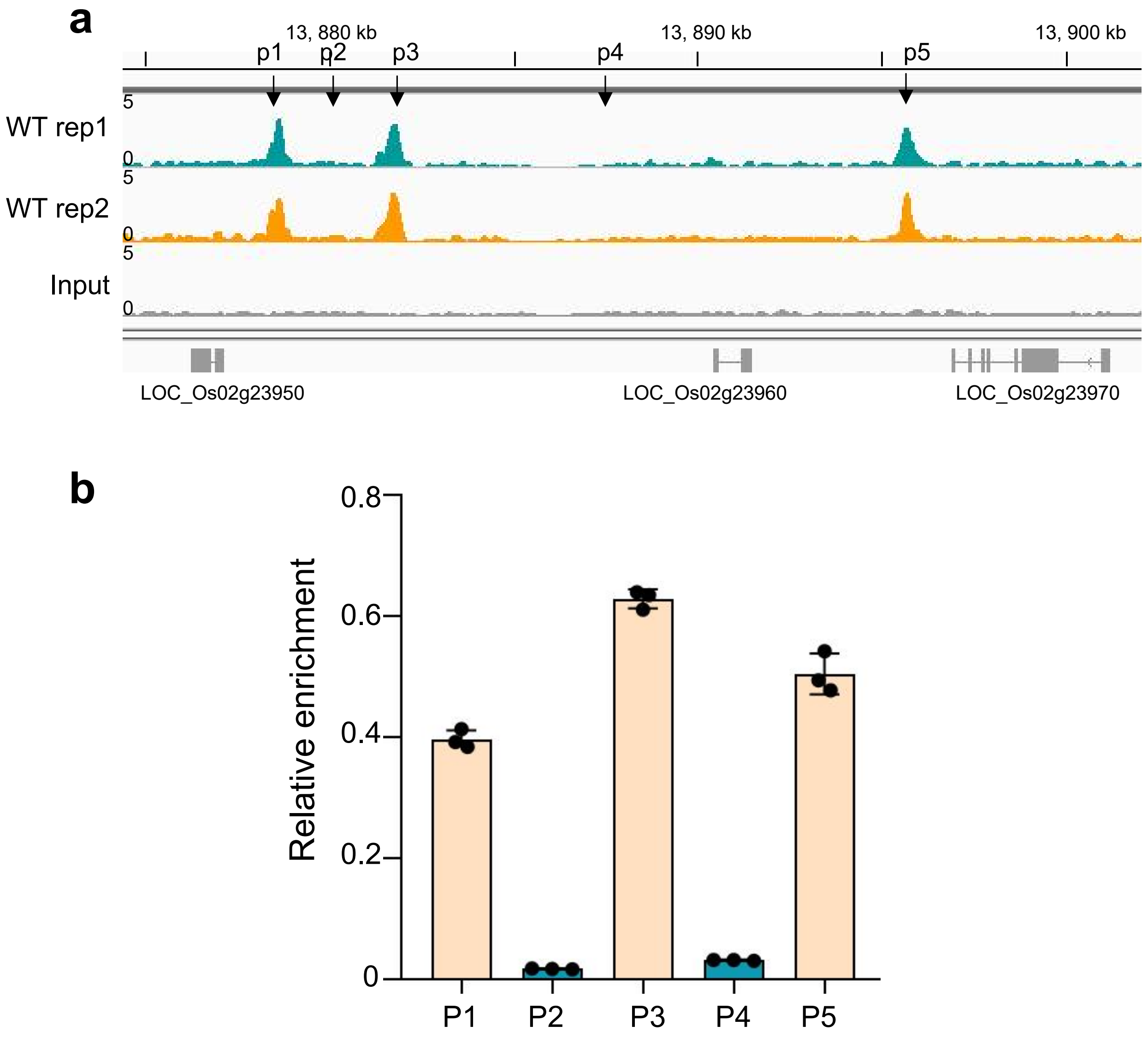

Supplementary Fig. S9. ChIP-qPCR validation of Histone Kbhb enriched regions (P1, P3, and P5) or depleted regions (P2 and P4). a IGV screenshots of selected regions (P1-P5) for tests. **b** ChIP-qPCR assay of the randomly selected regions (P1–P5). Bars are means ± SD from three biological replicates.

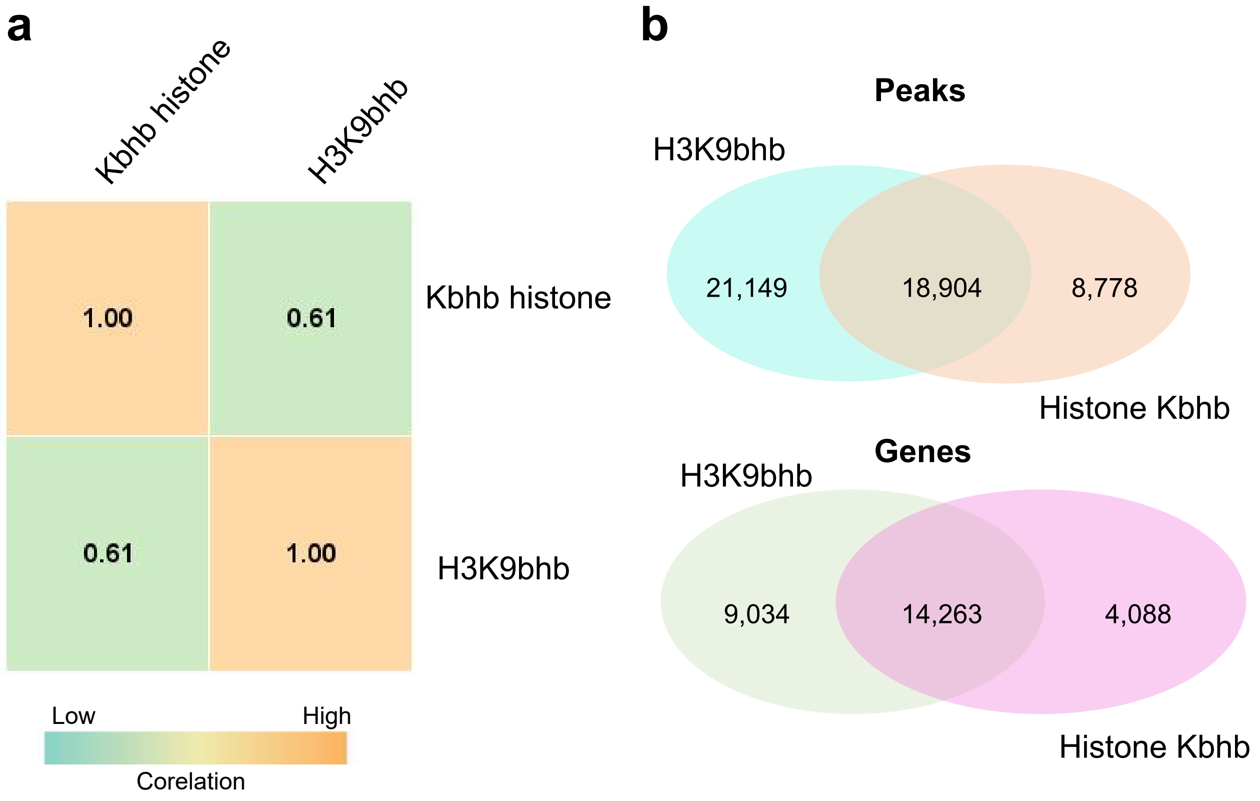

Supplementary Fig. S10. Comparative data analysis between H3K9bhb and histone Kbhb ChIP-seq datasets. a Correlation analysis between H3K9bhb and histone Kbhb ChIP-seq datasets. **b** Venn diagram showing the overlap of peaks and genes between H3K9bhb-marked and Kbhb-marked genes

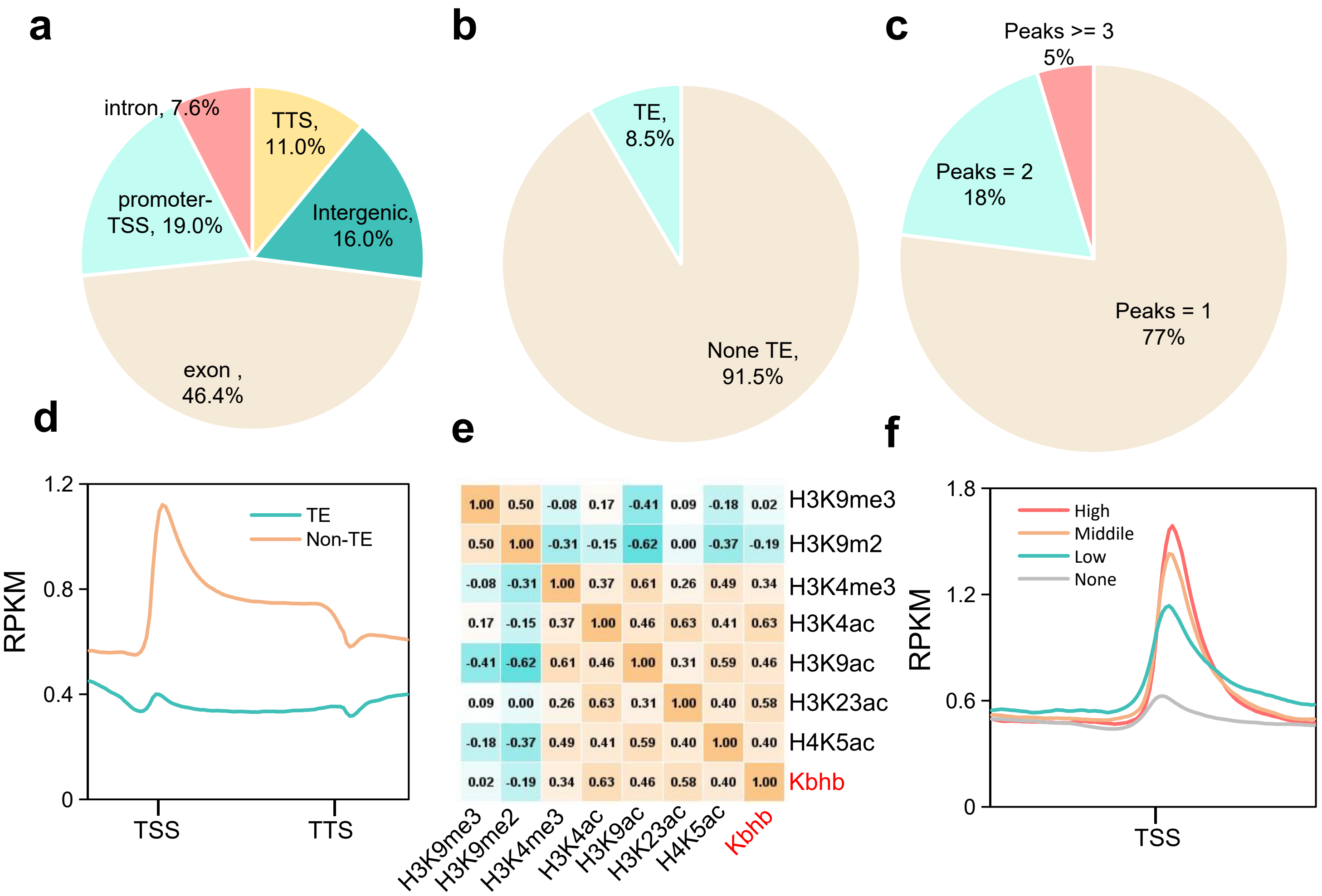

Supplementary Fig. S11. Characterization of histone Kbhb in rice. **a** Genomic distribution of histone Kbhb peaks in rice genome. **b** Pie chart of histone Kbhb marked TE and non-TE genes in rice. **c** Statistics on the number of Kbhb peaks per gene. **d** Metaplots of histone Kbhb in TE and None TE genes. **e** Correlation of histone Kbhb with other chromatin modifications. **f** Histone Kbhb signal intensity in genes grouped according to their RNA-seq counts into four categories: the top, middle, low, and bottom quartiles. TSS, transcriptional start site. TTS, transcriptional terminal site. TE, transposon elements.

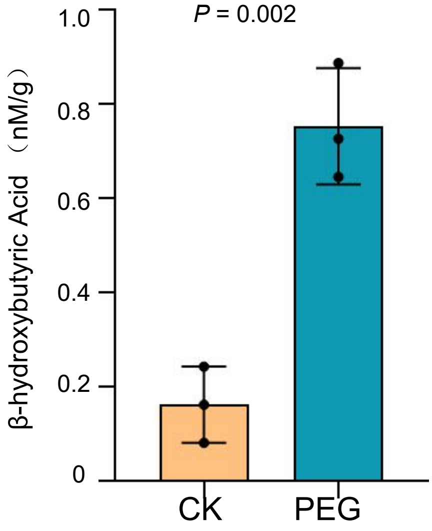

Supplementary Fig. S12. Assay of β-hydroxybutyrate levels in control (CK) and PEG-stressed rice plants. *P* values was calculated by two tailed student t test.

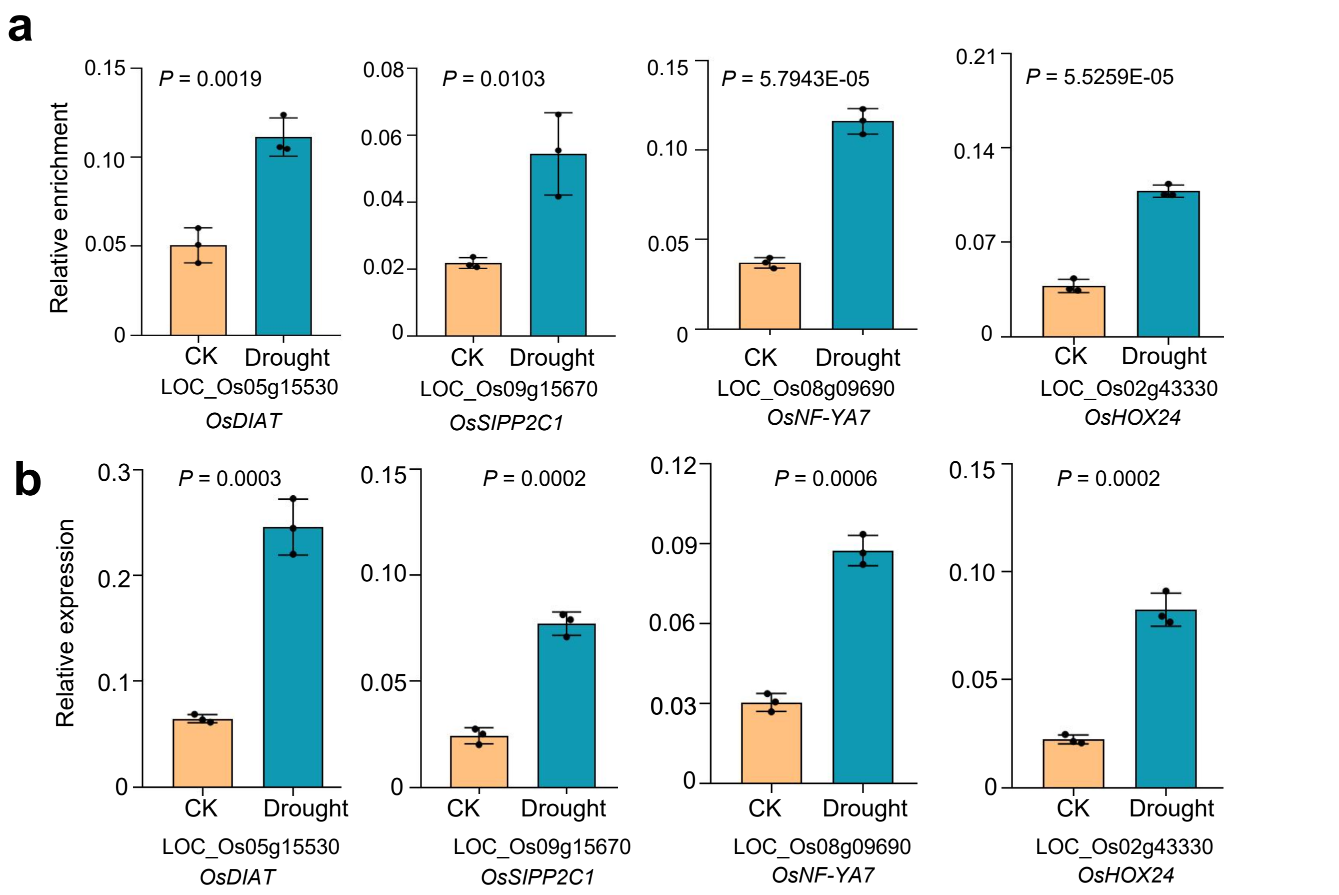

Supplementary Fig. S13. ChIP-qPCR (a) and RT-qPCR (b) analysis of histone Kbhb modification and gene expression levels of four selected drought resistance related genes in untreated (CK) and soil drought stressed plants. Significant differences between the groups were analyzed using a Student's *t* test.

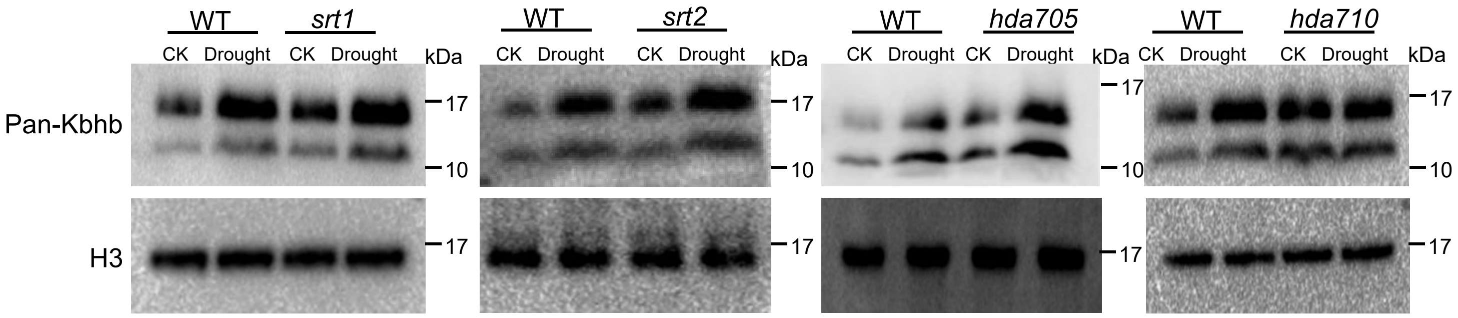

Supplementary Fig. S14. Assay of Histone Kbhb levels in wild type, *srt1*, *srt2*, *hda705,* and *hda710* rice plants under PEG and control (CK) conditions. Histone H3 was used as the loading control.

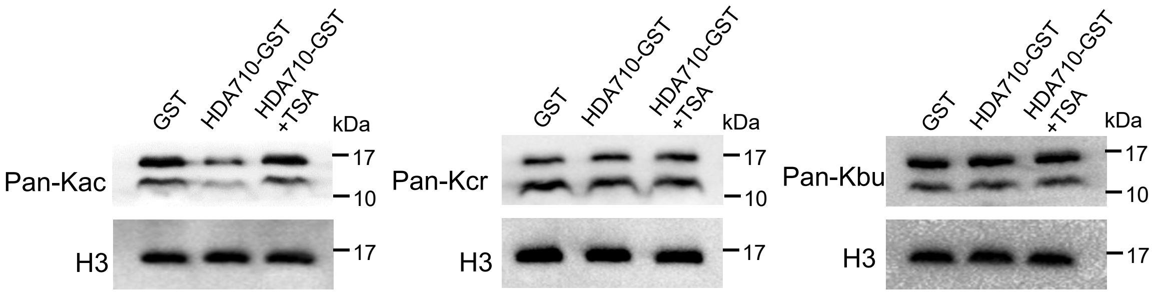

Supplementary Fig. S15. *In vitro* lysine de-acylase activity assay of HDA710 on histone acetylation (Kac), crotonylation (Kcr), and butyrylation (Kbu). Histone H3 was used as a loading control.

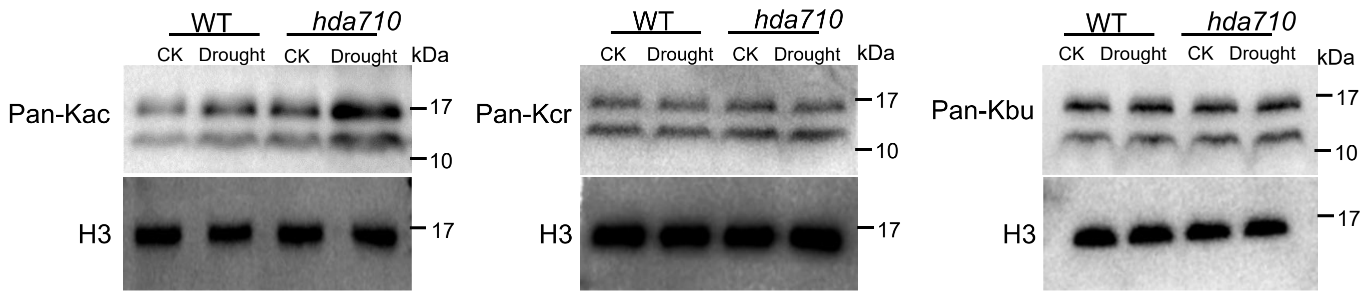

Supplementary Fig. S16. Assay of Histone Kac, Kcr, and Kbu levels in wild type and *hda710* rice plants under PEG and control (CK) conditions. Histone H3 was used as the loading control.

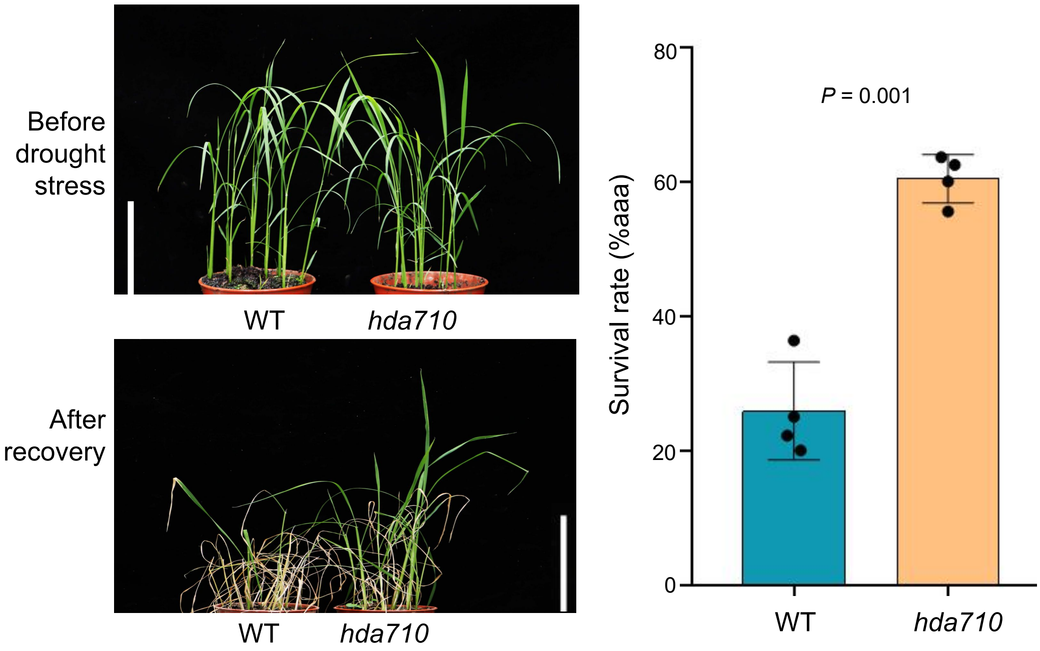

Supplementary Fig. S17. Survival rate of *hda710* plants and wild-type plants under soil drought stress. Seedlings were subjected to drought stress for 6 days, followed by a 10-day recovery period under normal growth conditions (25 °C). Bar = 10 cm. Significant differences between the groups were analyzed using a Student's *t* test.

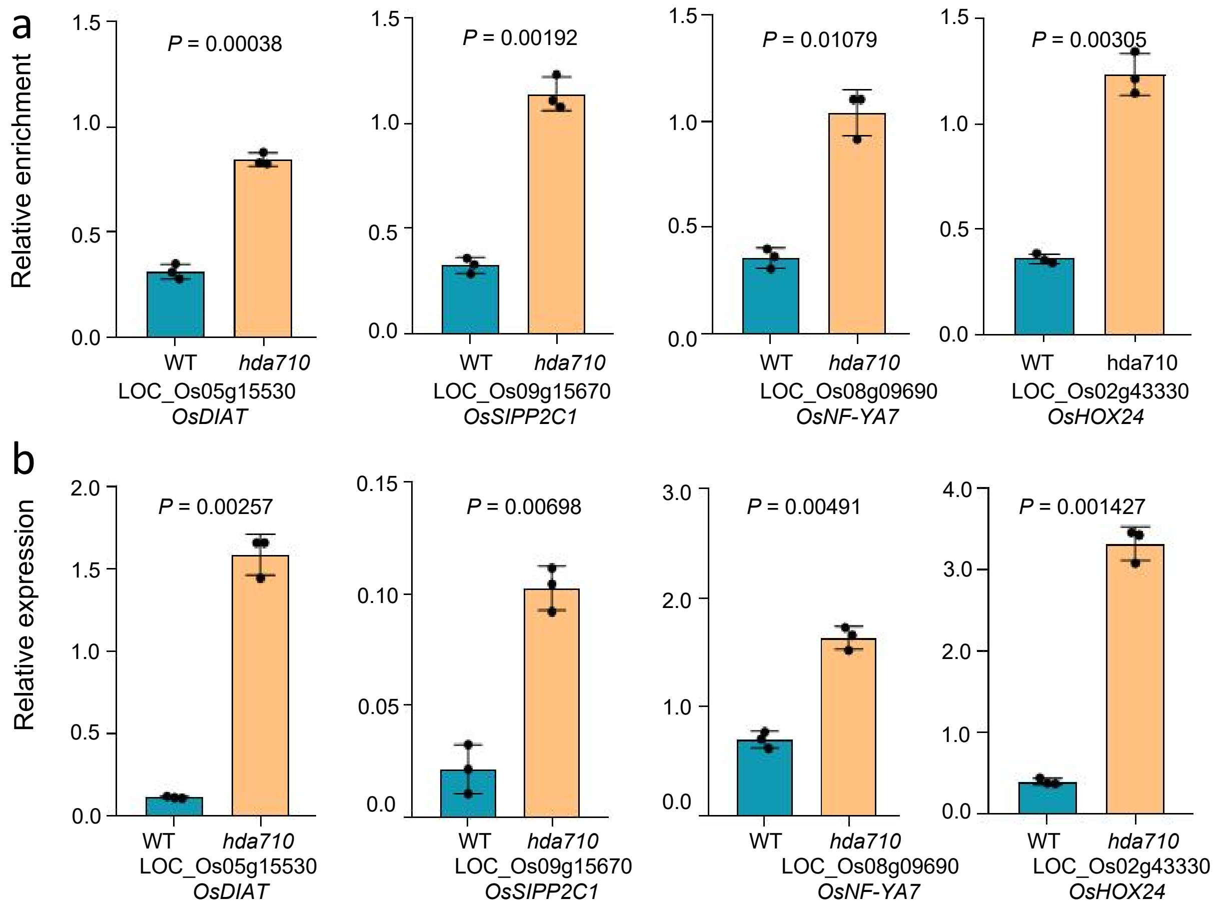

Supplementary Fig. S18. ChIP-qPCR (a) and RT-qPCR (b) analysis of histone Kbhb modification and gene expression levels of four selected drought resistance-related genes in *hda710* and wild type (WT) plants. Significant differences between the groups were analyzed using a Student's *t* test.
